# A high-quality chromosome-level genome assembly reveals genetics for important traits in eggplant

**DOI:** 10.1038/s41438-020-00391-0

**Published:** 2020-09-21

**Authors:** Qingzhen Wei, Jinglei Wang, Wuhong Wang, Tianhua Hu, Haijiao Hu, Chonglai Bao

**Affiliations:** Institute of Vegetable Research, Zhejiang Academy of Agricultural Sciences, Hangzhou, 30021 China

**Keywords:** Plant evolution, Structural variation

## Abstract

Eggplant (*Solanum melongena* L.) is an economically important vegetable crop in the Solanaceae family, with extensive diversity among landraces and close relatives. Here, we report a high-quality reference genome for the eggplant inbred line HQ-1315 (*S. melongena*-HQ) using a combination of Illumina, Nanopore and 10X genomics sequencing technologies and Hi-C technology for genome assembly. The assembled genome has a total size of ~1.17 Gb and 12 chromosomes, with a contig N50 of 5.26 Mb, consisting of 36,582 protein-coding genes. Repetitive sequences comprise 70.09% (811.14 Mb) of the eggplant genome, most of which are long terminal repeat (LTR) retrotransposons (65.80%), followed by long interspersed nuclear elements (LINEs, 1.54%) and DNA transposons (0.85%). The *S. melongena*-HQ eggplant genome carries a total of 563 accession-specific gene families containing 1009 genes. In total, 73 expanded gene families (892 genes) and 34 contraction gene families (114 genes) were functionally annotated. Comparative analysis of different eggplant genomes identified three types of variations, including single-nucleotide polymorphisms (SNPs), insertions/deletions (indels) and structural variants (SVs). Asymmetric SV accumulation was found in potential regulatory regions of protein-coding genes among the different eggplant genomes. Furthermore, we performed QTL-seq for eggplant fruit length using the *S. melongena*-HQ reference genome and detected a QTL interval of 71.29–78.26 Mb on chromosome E03. The gene *Smechr0301963*, which belongs to the *SUN* gene family, is predicted to be a key candidate gene for eggplant fruit length regulation. Moreover, we anchored a total of 210 linkage markers associated with 71 traits to the eggplant chromosomes and finally obtained 26 QTL hotspots. The eggplant HQ-1315 genome assembly can be accessed at http://eggplant-hq.cn. In conclusion, the eggplant genome presented herein provides a global view of genomic divergence at the whole-genome level and powerful tools for the identification of candidate genes for important traits in eggplant.

## Introduction

**Fig. 1:**
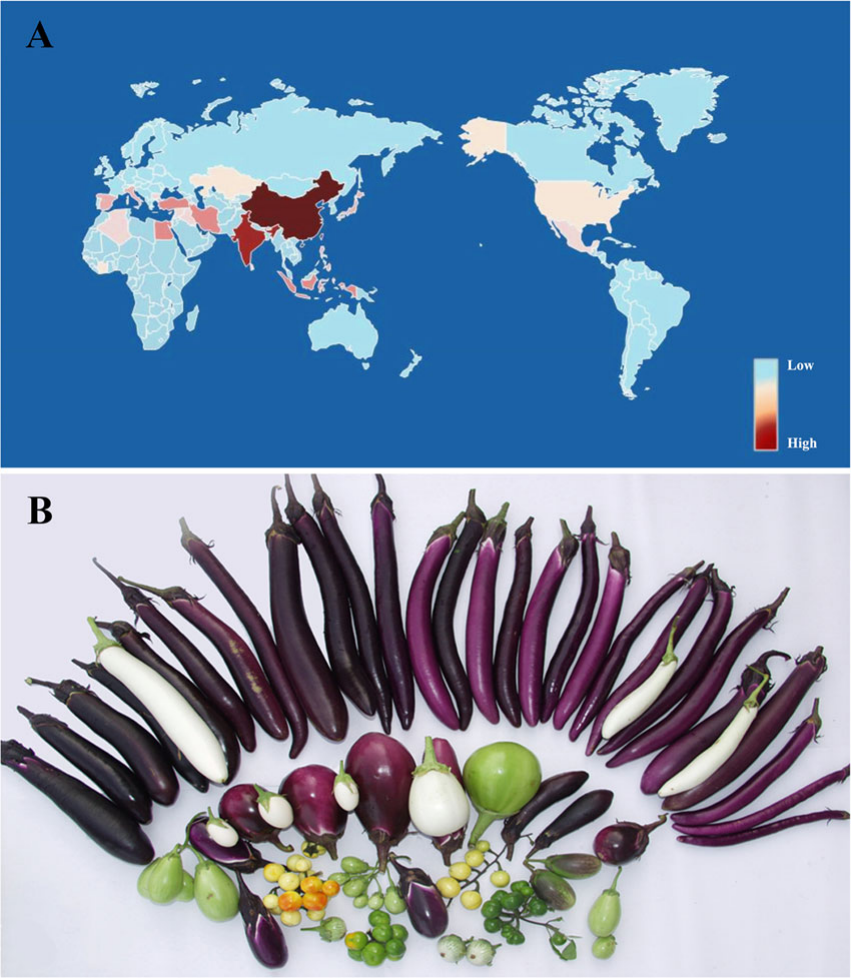
World production and diversity of eggplants. **a** Distribution of worldwide eggplant production according to FAOSTAT in 2018. **b** Diversity in fruit morphology among different eggplants

The large family Solanaceae contains over 3000 plant species that are adapted to a wide range of geographic conditions, including eggplant (*Solanum melongena*), tomato (*S. lycopersicum*), potato (*S. tuberosum*), tobacco (*Nicotiana tabacum*) and petunia (*Petunia inflata*). Asian eggplant (*S. melongena* L.), also known as brinjal or aubergine, is a vegetable crop widely grown across Southeast Asian, African, and Mediterranean countries^1^. Eggplant is the third most widely grown solanaceous vegetable after potatoes and tomatoes, with a global total production of ~54.08 million tons in 2018 (FAOSTAT; http://faostat3.fao.org). Approximately 90% of eggplants are produced in Asia, mainly in China and India, with Indonesia, Turkey, Egypt, the Philippines and Iran growing ~1% of the world’s total production^1^ (Fig. 1).

Unlike tomato and potato, which are both New World representatives of the genus *Solanum*^2^, eggplant is an Old World crop belonging to subgenus *Leptostemonum*^3^ (the “spiny solanums”). Two other *Solanum* species, Ethiopian/scarlet eggplant (*S. aethiopicum* L.) and African/Gboma eggplant (*S. macrocarpon* L.), are also called eggplants, and their fruits and leaves are used for food and medicine. There are obvious local preferences for eggplant fruits, which may be either elongated or round, with colors from dark purple to light green. The domestication history of eggplant has been under debate and presumably started in Africa, with radiation to Asia; however, relationships among the African species and their Asian relatives are not well resolved^4^. The two most commonly hypothesized regions of origin are India and southern China/Southeast (SE) Asia, which have equally old written records of eggplant use for ~2000 years^4^. Both regions have vastly diverse landraces, close wild relatives and candidate progenitors of eggplant. A recent study proposed that *S. insanum* is the wild progenitor, which split into an Eastern and Western group, with domesticates derived from the Eastern group^5^. Eggplants exhibit highly diverse variations in growth habits, biotic and abiotic resistance, and fruit and leaf morphology among local landraces and wild relatives. Identification of candidate genes/gene families controlling these differences will provide insight into the genetic mechanisms of agronomically important traits, as well as resources for eggplant breeding.

Genome sequencing is a powerful tool in plant genetics and genomics research. The genome of *Arabidopsis thaliana* was sequenced and published in 2000, representing the first plant genome. Since then, the development of genome sequencing technologies has resulted in multitude of plant genomes in recent years, including those of many horticultural crops^6–15^. Traditionally, the majority of research in *Solanum* crops has focused on potato and tomato, for which genomes have been published^9,10^. The first genome sequence of *S. melongena* was published in 2014, with 85,446 predicted genes and an N50 of 64 kb^13^. However, this draft assembly is not at the chromosome level and is highly fragmented, containing 33,873 scaffolds and covering only 74% of the eggplant genome. An improved *S. melongena* genome of the inbred line 67/3 using Illumina sequencing and single-molecule optical mapping was then published^16^. In addition, the genome of the African eggplant *S. aethiopicum*, a close relative of *S. melongena*, has also been published^17^. However, these eggplant genomes were all sequenced with next-generation sequencing (NGS) technologies using short reads, whereas genome sequence data derived from third-generation sequencing with long reads are still not publicly available. Here, we report a high-quality chromosome-level eggplant genome using next-generation Illumina sequencing and third-generation Nanopore sequencing combined with 10X genomic and Hi-C technologies, with a contig N50 of 5.26 Mb and a scaffold N50 of 89.64 Mb.

## Results

### Genome sequencing, assembly, and assessment

The genome size of the eggplant inbred line HQ-1315 is ~1205.25 Mb, with a heterozygosity rate of 0.15%, as assessed by k-mer analysis based on 93.33 Gb Illumina HiSeq data. The estimated proportion of repeat sequences was ~69.60%.

**Fig. 2:**
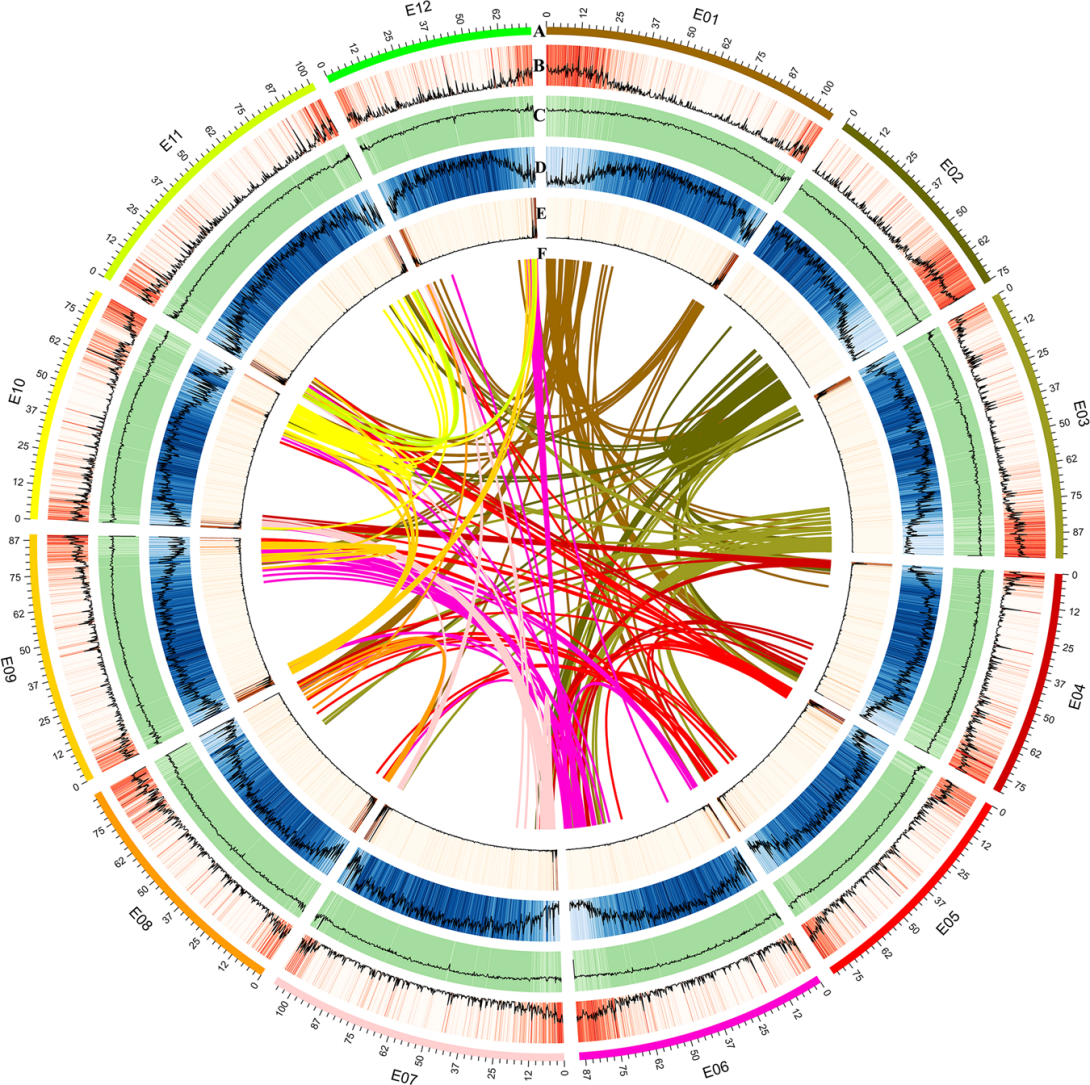
Circos diagram of different elements on eggplant chromosomes. E01-E12 refers to the 12 assembled chromosomes. **a** Assembled chromosomes (Mb). **b** Gene density. **c** GC content. **d** Transposon density. **e** Tandem repeat density. **f** Syntenic blocks

A high-quality eggplant genome (hereafter *S. melongena*-HQ) was assembled with a genome size of ~1.1 Gb and contig N50 of 5.26 Mb. We used a combination of Illumina HiSeq, Nanopore sequencing, and 10X Genomics sequencing technologies to sequence and assemble the eggplant genome; with the assistance of the Hi-C technique, a chromosome-level genome assembly was generated. A total of 114.45 Gb reads were obtained from Illumina HiSeq, including 93.33 Gb data for k-mer analysis and 21.12 Gb of additional read data, the average coverage of which was 94.96×; Nanopore sequencing generated 129 Gb data with 107.03× coverage. These data were used for preliminary assembly, producing a total contig length of 1159.53 Mb and a contig N50 of 5.71 Mb; the total scaffold length is 1159.53 Mb, with a scaffold N50 of 5.71 Mb. Then, we added ~113.46 Gb 10X Genomics data (~94.14×) for further assembly, resulting in a modified eggplant genome version with a contig length of 1,152.97 Mb and contig N50 of 5.75 Mb. The scaffold length is 1,157.36 Mb, with a scaffold N50 of 9.79 Mb, which is a 1.71-fold increase compared to the genome version by Hirakawa et al.^13^. Finally, with the assistance of 131.73 Gb Hi-C reads, the assembled scaffold N50 reached 89.64 Mb, with a final contig N50 of 5.26 Mb. Twelve pseudochromosomes with a total length of 1,173.14 Mb were obtained, accounting for 92.72% of the estimated eggplant genome (Fig. 2; Table 1). Detailed information on the stepwise assembly of the genome is shown in Table S1. The GC content in the eggplant genome is 35.94%, similar to that of *Arabidopsis*^18^ (36.06%), tomato^10^ (34.05%) and celery^15^ (35.35%) but lower than that of rice^19^ (43.57%) and tea plant^20^ (42.31%).

**Table 1:** Summary of the three published eggplant genomes

Genomes	*S. melongena*-HQ	*S. melongena*-67/3	*S. melongena*-NS
Size of assembly	1.07	1.14	0.83
Number of scaffolds	2263	10,383	33,873
Contig N50	5.26 Mb	16.7 kb	14.3 kb
Scaffold N50	89.64 Mb	2.9 Mb	0.065 Mb
Protein-coding genes	36,582	34,916	85,446
Annotated BUSCO genes	2190 (94.2%)	1332 (96.9%)	1028 (74.8%)
Repeats (%)	70.09	73	–
GC content (%)	35.94	36	35.7

The quality of the eggplant genome assembly was further assessed (Supplementary Fig. S1). The alignment rate of all short reads to the genome was ~99.48%, covering 91.24% of the genome. The heterozygous and homozygous SNP ratios were calculated to be 0.0253% and 0.0014%, respectively, indicating a high single-base accuracy rate for the genome assembly. The integrity of the assembled genome was assessed by the Core Eukaryotic Genes Mapping Approach (CEGMA); 237 genes were assembled from 248 core eukaryotic genes (CEGs), accounting for 95.56% of the total and reflecting that the sequence assembly was relatively complete. The statistical results of BUSCO evaluation of the eggplant genome showed that 2,190 homologous single-copy genes were assembled and that 94.2% of all single-copy genes were assembled.

### Genome annotation

For the annotation of the eggplant genome, we used a combination of gene prediction strategies, including de novo, homology, and transcriptome-based predictions. RNA from five different tissues, including root, stem, leaf, flower and fruit, was extracted for next-generation transcriptome sequencing and full-length transcriptome sequencing. A total of 36,582 coding genes were predicted, with an average of 4.31 exons per gene and an average transcript length of 4095.69 bp. Repetitive sequence annotation results showed that 70.09% of the eggplant genome is repeat sequences, with a size of 811.14 Mb. Most of the repeat sequences are long terminal repeat (LTR)-type retrotransposons, which account for 65.80%; 1.54% is the long interspersed nuclear element (LINE) type, and DNA transposons account for only 0.85%. In addition, 5929 noncoding RNAs were detected in the eggplant genome, including 268 miRNAs with an average length of 127.81 bp as well as 2549 tRNAs, and 554 snRNAs (Supplementary Table S2).

### Evolution of the *S. melongena* genome

**Fig. 3:**
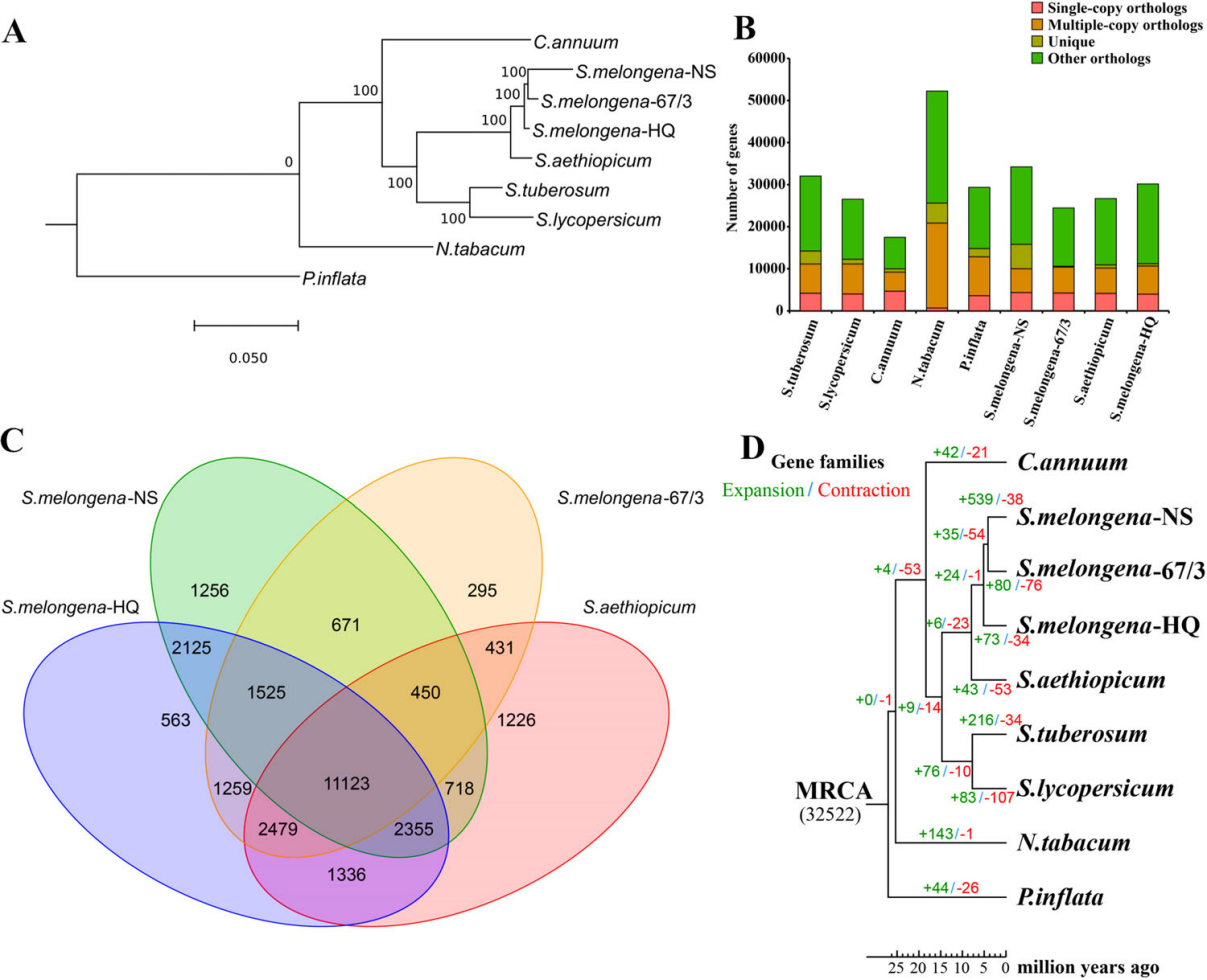
Comparative analysis of the *S.melongena*-HQ genome. **a** Phylogenetic tree of 9 sequenced Solanaceae genomes. **b** Distribution of genes in different species. The horizontal axis indicates the analyzed genome, and the vertical axis indicates the number of corresponding genes. Pink represents the number of single-copy orthologs. Orange represents multiple-copy orthologs, olive green the unique genes of the corresponding genome, and green the number of other orthologs. **c** Venn diagram of the common and unique gene families among different eggplant genomes. **d** Gene family expansion and contraction in the 9 Solanaceae genomes. The green numbers indicate expanded gene families, and the red numbers represent contracted gene families

A total of 9 sequenced Solanaceae genomes were analyzed to reveal the evolution of the eggplant genome, including *Nicotiana tabacum*, *Capsicum annuum*, *Petunia inflata*, *S. tuberosum*, *S. lycopersicum*, *S. aethiopicum*, *S. melongena*-HQ, and two other *S. melongena* genomes, *S. melongena*-NS^13^ and *S. melongena*-67/3^16^. Phylogenetic analysis indicated that eggplant is closer to potato and tomato than pepper (Fig. 3a), diverging from the common ancestor ~14.4 Mya (Fig. S2). The group of three *Solanum* species (eggplant, potato and tomato) is sister to pepper, diverging ~18.5 Mya. Among the different eggplants, *S. melongena*-HQ and its close relative *S. aethiopicum* diverged from a common ancestor ~2.4 Mya (Fig. S2). Moreover, *S. melongena*-HQ is more closely related to the European eggplant variety *S. melongena*-67/3 than the Japanese eggplant cultivar *S. melongena*-NS, all of which are distant from *S. aethiopicum* (Fig. 3a).

There were 32,529 gene families in total according to clustering results. Among the nine genomes, 6087 gene families are common, of which 463 single-copy gene families are common to each genome (Fig. 3b). The corresponding clustering results for *S. melongena*-NS, *S. melongena*-67/3, *S. aethiopicum,* and *S. melongena*-HQ were extracted to draw a Venn diagram, which showed that the four eggplant genomes have 11,123 genes (Fig. 3c). Compared with other eggplants, *S. melongena*-NS has the most unique genes (1,256 genes), followed by *S. aethiopicum* with 1226 unique genes; *S. melongena*-67/3 has only 295 unique genes. In addition, *S. melongena*-HQ has a total of 563 accession-specific gene families containing 1009 genes (Fig. 3c, Supplementary Table S3). We performed GO and KEGG enrichment analyses on accession-specific gene families of *S. melongena*-HQ (Supplementary Table S3) and found them to be mainly involved in the processes of metabolism, biosynthesis and modification of proteins/nucleic acids.

**Fig. 4:**
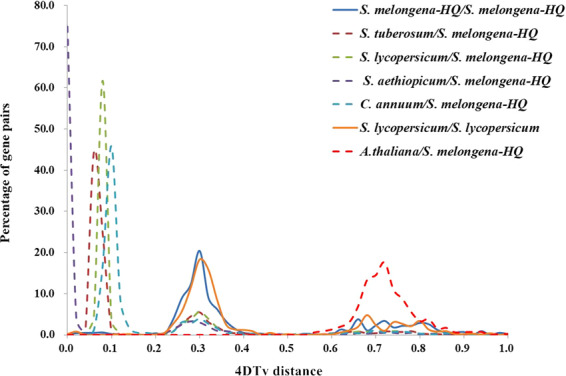
Distribution of 4DTv distances. The *x*-axis indicates the 4DTv distance. The *y*-axis indicates the percentage of gene pairs

Whole-genome duplication (WGD) events in the *S. melongena*-HQ genome were detected based on the rate of fourfold degenerative third-codon transversion (4DTv) of paralogous gene pairs among *S. melongena*-HQ, *A. thaliana* and four other Solanaceae species. As illustrated in Fig. 4, *A. thaliana* and *S. melongena*-HQ had one peak value at ~0.72, indicating an ancient WGD before the divergence of asterids and rosids. *S. melongena*-HQ had only one WGD event common to Solanaceae species at ~0.30, whereas there was no recent WGD after species differentiation. Among Solanaceae crops, *S. melongena*-HQ first diverged from pepper at ~0.1, followed by tomato at ~0.08, and then *S. tuberosum* at ~0.06. The two eggplants *S. aethiopicum* and *S. melongena*-HQ diverged from each other quite recently compared with other species.

### Expansion and contraction of gene families

The 9 sequenced Solanaceae genomes were analyzed to reveal the dynamics of gene family evolution in the eggplant genome. A total of 32,522 most recent common ancestor (MRCA) gene families were found (Fig. 3d). Compared with their ancestors six gene families expanded and 23 gene families contracted in *S. melongena* and *S. aethiopicum*. Among the different eggplant genomes, *S. melongena*-NS has 539 gene families that significantly expanded and 38 gene families that contracted, whereas *S. melongena*-67/3 has 80 expanded gene families and 76 contracted gene families. *S. melongena*-HQ has 73 expanded gene families, including 892 genes, and 34 contracted gene families, including 114 genes (Fig. 3d, Supplementary Table S4). The expanded and contracted genes were also annotated by GO and KEGG analyses (Supplementary Table S4). The KEGG pathway plant-pathogen interaction showed the most contracted genes (25 genes), which may be related to reduced resistance in cultivated eggplant.

### Comparative genomic analysis

**Fig. 5:**
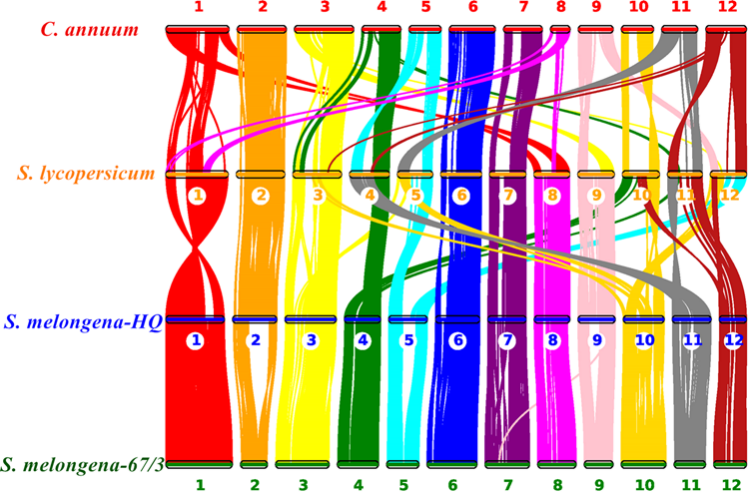
Synteny analysis of genes in *C. annuum*, *S. lycopersicum*, *S. melongena-*67/3, and *S. melongena*-HQ. The numbers indicate the corresponding chromosomes in each species

Synteny analysis showed that the *S. melongena*-HQ genome exhibits high collinearity with that of *S. melongena*-67/3, with a total of 19,620 gene pairs and 178 syntenic blocks. Chromosome E01 in these two eggplant genomes is in the same direction but inverted compared with tomato chromosome 1. There is one missing block in *S. melongena*-67/3 chromosome E02, which exists between *S. melongena*-HQ and tomato and between tomato and pepper. Similar missing segments were also found for corresponding chromosomes 5 and 9. Chromosomes 4, 5, 10, 11, and 12 have undergone more complex chromosome rearrangements, such as translocations and inversions, during evolution among eggplant, tomato and pepper, as reflected by an increased number of syntenic blocks. We identified a total of 18,337 gene pairs and 151 syntenic blocks between *S. melongena*-HQ and tomato. *S. melongena*-HQ chromosome E04 was partly aligned to tomato chromosomes 4, 10 and 11; some of the genes on *S. melongena*-HQ chromosome E05 were aligned to tomato chromosome 12. Genes from *S. melongena*-HQ chromosome E10 were aligned to *S. lycopersicum* chromosomes 3, 5 and 12. Similar collinearity was also detected among the genes from corresponding chromosomes 11 and 12 between *S. melongena*-HQ and *S. lycopersicum* (Fig. 5). Pairwise comparisons are presented in Supplementary Figs. S3–S5.

Although the overall genome lengths of *S. melongena*-HQ and *S. melongena*-67/3 are not significantly different, the length of each chromosome differ significantly (Table 2). The total sizes of the two eggplant genomes are 1073.14 and 1142.80 Mb, respectively, with a total size difference of 69.66 Mb. The largest difference is with regard to chromosome E09; the length of E09 in *S. melongena*-HQ is 89.64 Mb, whereas that of *S. melongena*-67/3 is only 36.10 Mb, with a difference of 53.54 Mb. The smallest difference was found for E03, with a difference of only 0.30 Mb, followed by E02, with a difference of only 7.92 Mb. The length of E05 in *S. melongena*-HQ is 37.74 Mb longer than that in *S. melongena*-67/3, and the length of *S. melongena*-HQ E07 is 35.59 Mb shorter than that of *S. melongena*-67/3. The differences in the lengths of other chromosomes, E04, E08, E06, E10, E11, and E12, are between 19.30 and 28.93 Mb. Despite the minor differences in total genome size between the two assembled eggplant genome versions themselves, the differences in chromosome length between the two assembled versions are significant. This result may have been caused by different sequencing technologies (second vs third generation) and assembly strategies (linkage map vs Hi-C).

**Table 2:** Comparison of chromosome lengths between *S. melongena*-HQ and *S. melongena*-67/3

Chr. No.	Chromosome length of *S. melongena*-HQ	Chromosome length of *S. melongena*-67/3	Difference
E01	106.64	136.53	−29.90
E02	75.42	83.34	−7.92
E03	96.71	97.01	−0.30
E04	80.28	105.67	−25.39
E05	81.59	43.85	37.74
E06	89.68	108.97	−19.30
E07	106.79	142.38	−35.59
E08	86.83	109.58	−22.74
E09	89.64	36.10	53.54
E10	84.17	106.64	−22.48
E11	101.22	72.29	28.93
E12	74.17	100.42	−26.25
TOTAL	1073.14	1142.80	−69.66

**Fig. 6:**
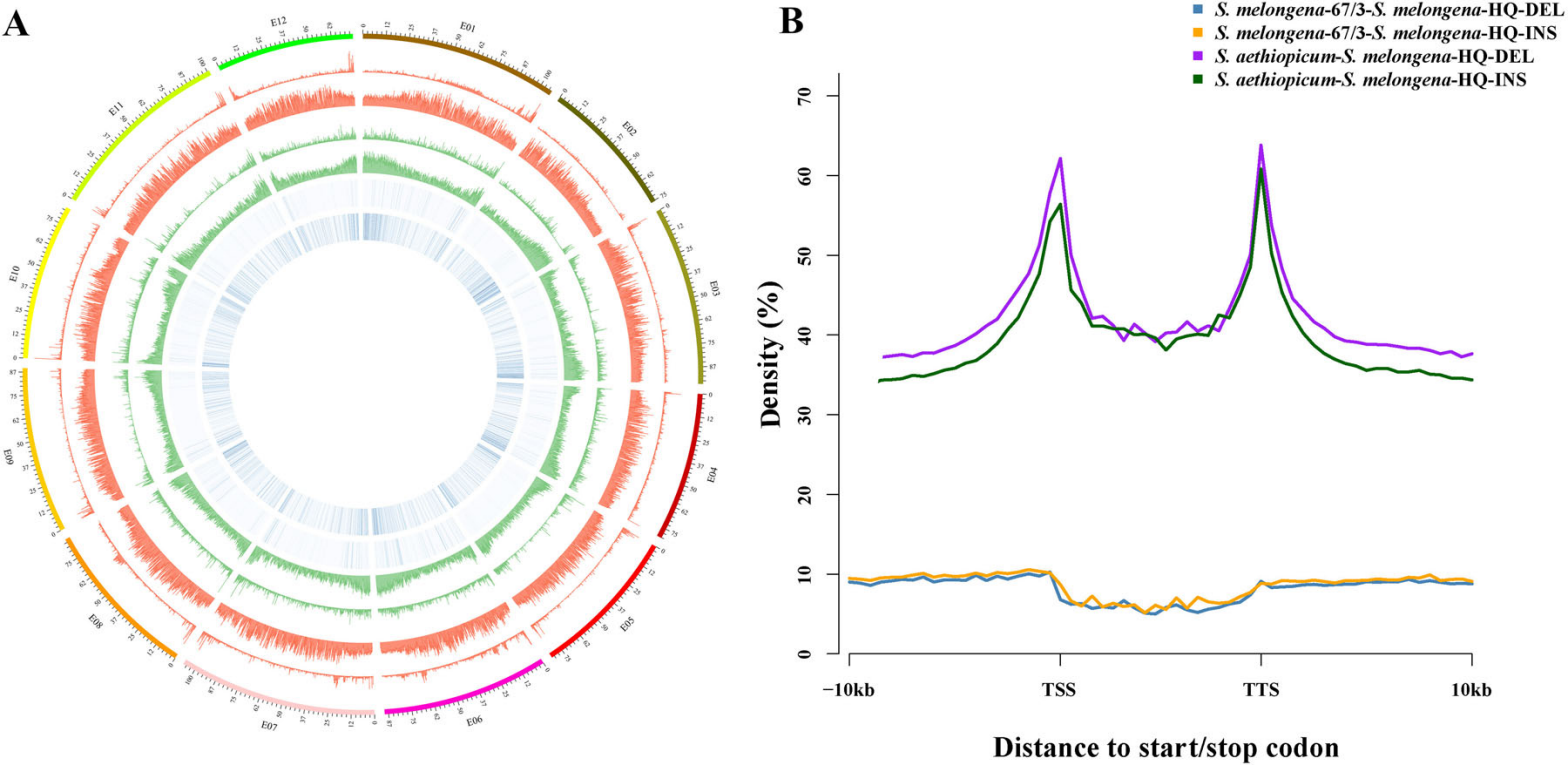
The variation analysis among *S. melongena*-HQ, *S. melongena*-67/3 and *S. aethiopicum* **a** Asymmetric SV accumulation among different eggplants. The tracks (from outside to inside) indicate chromosomes, SNP density, indel density, and percentage of SV length. **b** SV variation percentages in potential regulatory regions of protein-coding genes. The horizontal axis indicates up- and downstream gene regions, and the vertical axis indicates the variation percentage. Pink represents the number of single-copy orthologs. Purple and green lines indicate SV deletions and insertions between *S. melongena*-HQ and *S. aethiopicum*, respectively. Blue and yellow lines indicate SV deletions and insertions between *S. melongena*-HQ and *S. melongena*-67/3, respectively

We then compared *S. melongena*-HQ with two previously sequenced eggplant genomes, those of European eggplant *S. melongena*-67/3 and African eggplant *S. aethiopicum*, to investigate genomic divergence among them (Fig. 6a). Three types of variations were analysed, including single-nucleotide polymorphisms (SNPs), insertions/deletions (indels) and structural variants (SVs). We detected 2,189,112 SNPs, 512,849 indels, and 741 large SVs between *S. melongena*-HQ and *S. melongena*-67/3. In contrast, 22,092,994 SNPs, 1,988,560 indels, and 7,362 large SVs were identified between *S. melongena*-HQ and *S. aethiopicum*. Between *S. melongena*-HQ and *S. melongena*-67/3, the 512,849 indel mutations involve 14,756 genes, which were annotated using GO and KEGG (Supplementary Table S5). The 741 SVs correspond to 211 genes, among which 60 were functionally enriched by GO analyses (Supplementary Table S5). For *S. melongena*-HQ and *S. aethiopicum*, 3,066 genes are associated with large SVs, among which 1,370 and 350 genes were functionally enriched according to GO and KEGG analysis, respectively (Supplementary Table S6). There are 90 genes involved in antibiotic biosynthesis networks according to the KEGG enrichment results, and 16 genes related to the citrate cycle (TCA cycle). It has been proposed that the African eggplant *S. aethiopicum* has better disease resistance and drought tolerance than cultivated *S. melongena*-HQ^17^. Therefore, these genes will provide valuable resources for resistance improvement in eggplant breeding.

We further investigated SV abundance in potential regulatory regions of protein-coding genes; different types of indel variation suggest different patterns of SV accumulation (Fig. 6b). There were more deletions than insertions between *S. melongena*-HQ and *S. aethiopicum*. However, insertions and deletions between the two *S. melongena* genomes were similar in both coding and noncoding areas, with the two lines basically coinciding. Higher insertion-deletion variations were observed in transcription start site (TSS) and transcription terminal site (TTS) regions of *S. melongena*-HQ and *S. aethiopicum*, and the variation in the gene coding regions was found to be slightly higher than that in noncoding regions. In contrast, variations in coding regions were lower than those in noncoding region between cultivated eggplants.

### NBS gene family and transcription factor analysis

Nucleotide-binding site-leucine-rich repeat (NBS-LRR) proteins constitute the largest family of resistance (R) proteins and play significant roles in defense against pathogens. The NBS protein family was systematically analysed in five plants of the Solanaceae family. In *S. melongena*-HQ, 301 NBS genes were identified as involved in seven types (Table 3; Supplementary Table S7), whereas only 250 genes were identified in *S. melongena*-67/3 as involved in eight types. *S. aethiopicum* has outstanding resistance to various pathogens, including *Fusarium*, *Ralstonia* and *Verticillium*^21,22^, with 436 NBS genes involved in ten types. Accordingly, *S. aethiopicum* has been routinely used to improve disease resistance in *S. melongena*. *S. lycopersicum* was found to possess 223 NBS genes.

**Table 3:** Summary of the NBS gene family

Species	NBS	NBS-LRR	LRR-NBS	LRR-NBS-LRR	TIR-NBS	TIR-NBS-LRR	Others	Class No.	Total No.
*S. melongena-*HQ	133	114	4	9	10	30	1	7	301
*S. melongena-*67/3	111	82	4	9	8	34	2	8	250
*S. aethiopicum*	219	145	11	10	16	31	4	10	436
*S. lycopersicum*	81	100	9	8	6	17	2	8	223

In terms of transcription factors, for *S. melongena-*HQ, a total of 1970 transcription factors divided into 64 categories, the top three of which were APETALA2/ethylene responsive factor (AP2/ERF, 150), cysteine 2-histidine 2 type zinc finger gene (C2H2, 137) and basic helix-loop-helix (bHLH, 135) were identified. The v-myb avian myeloblastosis viral oncogene homolog superfamily (MYB) has 127 transcription factors. Detailed information on the number and gene sequences of each transcription factor, including *S. melongena*-67/3, *S. aethiopicum* and *S. lycopersicum*, is shown in Supplementary Table S8.

### Candidate gene identification for fruit length and QTL hotspots in eggplant

**Fig. 7:**
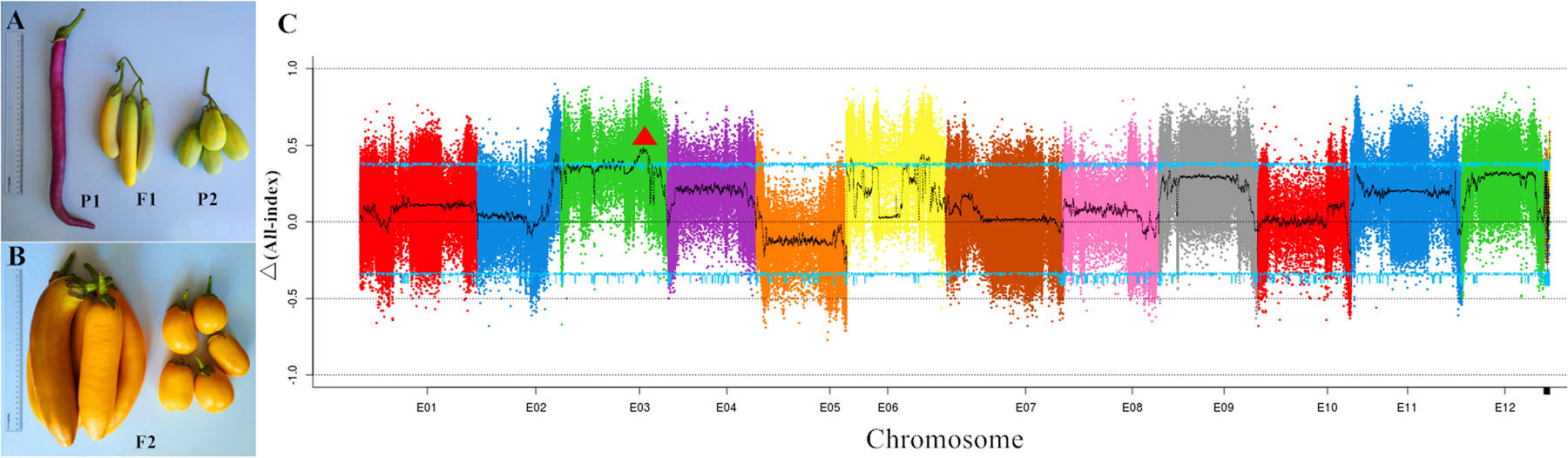
QTL analysis of fruit length trait. **a** Fruits of P_1_, P_2_, and F_1_. **b** F_2_ individuals with extreme fruit lengths. **c** Distribution of Δ (All-index) on chromosomes. The light blue line indicates the results of a replacement test with 1000 replicates; the 95% confidence level was selected as the screening threshold

Eggplants display extensive variations in fruit morphology among landraces and wild relatives. There are obvious local market preferences for fruit shape (i.e., oval, round or linear) according to different consuming habits; thus, the fruit length, diameter and shape index of eggplants show significant differences (Fig. 1). The immature fruits of HQ-1315 are generally ~35 cm in length and ~3 cm in diameter, and it is a long (elongated type) eggplant. An F_2_ population containing 129 individuals was obtained from a cross between HQ-1315 (P_1_) and the short round eggplant 1815 (P_2_; Fig. 7). Bulked segregant analysis (BSA) and quantitative trait locus (QTL) analysis on eggplant fruit length were then conducted using the *S. melongena*-HQ genome (Fig. 7). F_2_ plants with extremely long and short fruits were selected and pooled for genome sequencing. Resequencing P_2_ generated 23.41 Gb of data, and sequencing of the two extreme pools yielded 41.52 Gb for the extreme long pool and 40.05 Gb for the extreme short pool. The average length (L), diameter (D), and fruit shape index (L/D) of three fruits from each F_2_ individual were measured to determine the value for the individual plant (Supplementary Table S9). Based on genotyping results, a total of 1,019,131 SNPs and 116,676 indel markers showed homozygous differences between the two parents, and the index of the markers in the two progeny pools compared to those of the parents were analyzed and calculated. According to the Δ(All-Index) value, a QTL interval for fruit length was determined within 71.29–78.26 Mb on eggplant chromosome E03 (99% confidence interval) (Fig. 7). Combined with the genetic mapping results of our previous study, Marker2384739 and Marker2387171 are linked to QTL *FS3.1*, the physical locations of which are 77.62 and 79.77 Mb respectively. As suggested by the eggplant-tomato synteny relationship, genes controlling fruit size in tomato are likely to have similar functions in determining eggplant fruit size. We obtained a total of 11 genes homologous to regulators of fruit size on eggplant chromosome E03 via homology comparison. Among them, three genes are within or adjacent to the QTL region on E03: *Smechr0301760* (72.91 Mb), *Smechr0301963* (78.39 Mb) and *Smechr0302217* (82.30 Mb). *Smechr0301760* is a potentially orthologous gene of the *cell number regulator* (*CNR*) gene family, whereas *Smechr0301963* and *Smechr0302217* are potentially orthologous genes of the *SUN* gene family. According to the results of QTL-seq and genetic mapping, we predict that *Smechr0301963* is a key candidate gene for regulating eggplant fruit length. Moreover, 7 homologs of fruit size-related genes are distributed within 89.89–95.48 Mb region, and they may also play potential roles in controlling fruit size.

**Fig. 8:**
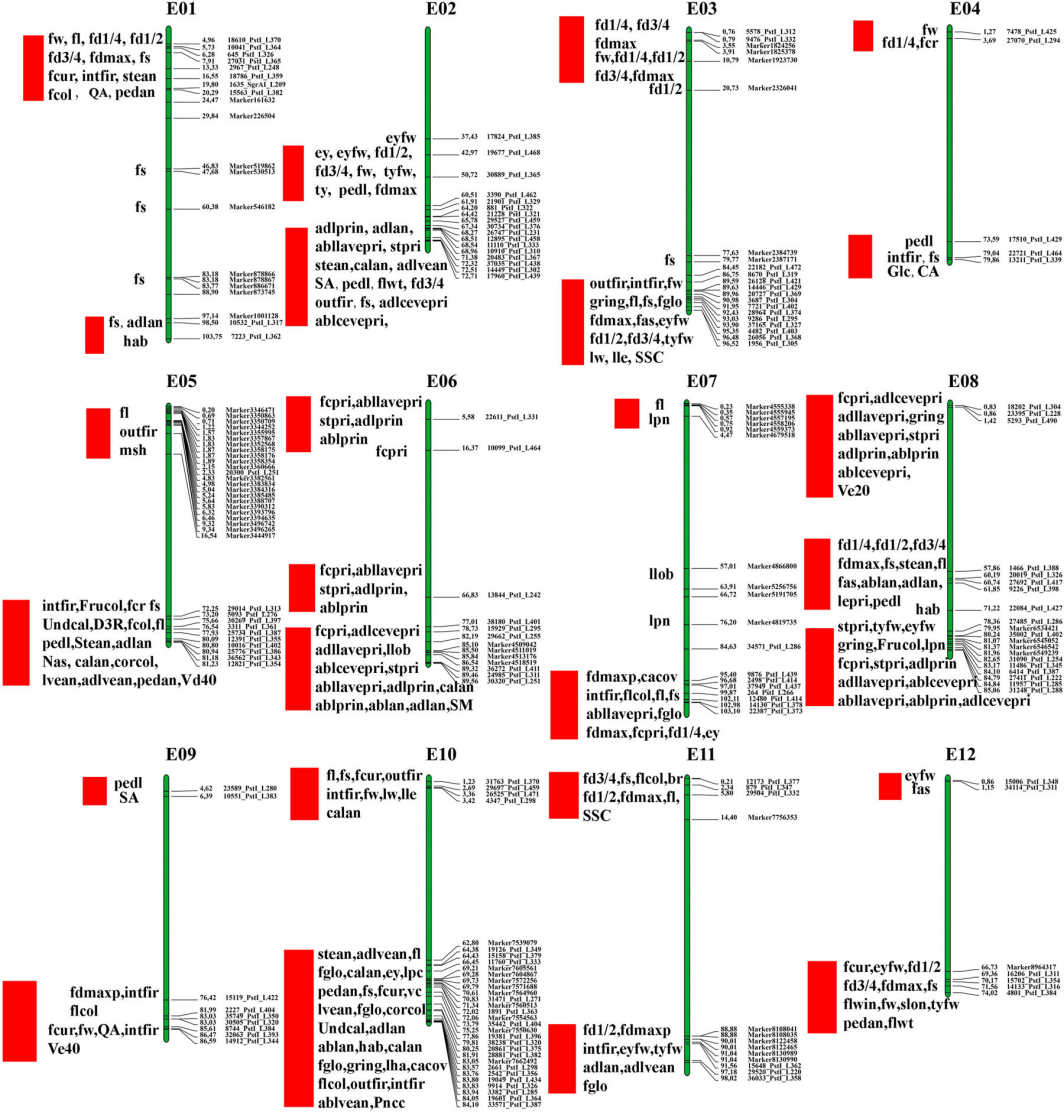
Distribution of QTL hotspots on 12 eggplant chromosomes. The physical locations and marker names are on the right of each chromosome; names of associated traits are on the left of each chromosome. Red rectangles represent QTL hotspots. The full names of the traits involved and corresponding markers are shown in Supplementary Table S10

Based on the QTL results of previous studies and the available marker sequence information, we anchored these markers to our latest reference genome to investigate QTL hotspots in eggplant^23–30^. A total of 210 linkage markers related to 71 traits, including fruit-related traits (i.e., fruit size and color), leaf morphology, and nutrient components, were anchored (Fig. 8, Supplementary Table S10). Except for the linkage markers for *Fusarium* resistance in Miyatake et al.^29^, most of the markers were mapped to physical locations on corresponding chromosomes. We summarized the regions with clustered linkage markers or traits and finally obtained 26 QTL hotspots, with two to three on each chromosome.

### Eggplant Genome Database

**Fig. 9:**
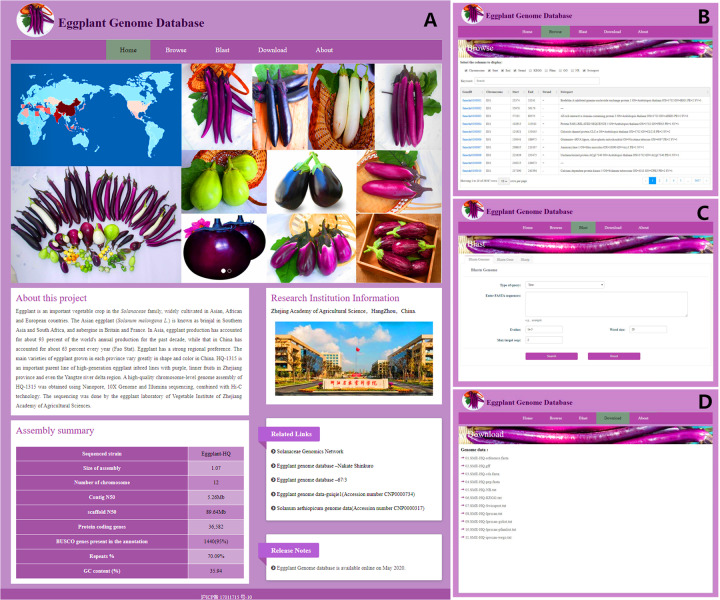
An overview of Eggplant Genome Database. **a** Homepage. **b** Browse module. **c** Blast module. **d** Download module

We constructed an advanced, intuitive, and user-friendly Eggplant Genome Database using genome assembly and annotation data (Fig. 9). Eggplant Genome Database consists of three main modules. The browse module has links to information for 36,582 genes, including start/end locations and chromosome information. KEGG, Pfam, GO, NR, and Swiss-Prot database annotation information can be easily accessed by clicking the gene ID, as can the coding sequence (CDS) and protein sequence information corresponding to each gene. The BLAST module aligns sequences to the genome, gene, and protein databases to obtain the required information for users. The eggplant genome assembly, as well as genome gff, CDS, protein, and other data files, can be downloaded using the download module. Eggplant Genome Database provides access to various types of data, allowing researchers and breeders to browse, search, and download information for genomics studies and breeding. The online database can be accessed at http://eggplant-hq.cn/.

## Discussion

Genome sequencing technologies have undergone tremendous improvement during the past decades, resulting in substantial advances in the availability of plant genomes. Since the publication of the first plant genome, *Arabidopsis thaliana*, using whole-genome shotgun sequencing, over 200 plant genomes have been published^31^ (www.plabipd.de). However, genome sequencing of plant species with large genome sizes and high repetitive sequence contents remains difficult^32^. Compared with the short reads produced by NGS technologies, long reads with kilobase-length DNA fragments are extremely efficient in resolving repetitive regions and facilitating genome assembly. Several new technologies have been developed based on long reads, such as synthetic long reads, long PacBio reads, and optical mapping, and these methods have been applied to *Arabidopsis*^33^, tomato (3.0 genome release; www.solgenomics.net) and maize^34^. Nevertheless, long-read sequencing technologies are still costly and rely on the previous extraction of high-quality DNA. Oxford Nanopore is a recently developed long-read sequencing technology that can greatly reduce the sequencing costs and generate gigabases of sequence data from a single flow cell^35^. Hi-C proximity ligation is another driving technology that may help in the assembly of fragmented plant genomes at the chromosome level^36^. In the present study, we combined 114.45 Gb Illumina short reads with 129 Gb long reads from Nanopore sequencing and ~113.46 Gb 10X Genomics data to generate a high-quality eggplant genome, with a contig N50 of 5.26 Mb and a scaffold N50 of 89.64 Mb. With the assistance of 131.73 Gb Hi-C data, 12 eggplant pseudochromosomes were obtained, with a total size of ~1.07 Gb, covering 92.72% of the eggplant genome. Both contig N50 and scaffold N50 were significantly improved compared with those of previously published *S. melongena* genomes^13,16^. The number of scaffolds obtained was 10,383 for *S. melongena*-67/3 and 33,873 for *S. melongena*-NS; we assembled 2,263 scaffolds. A total of 36,582 protein-coding genes were detected in the present study, similar to the ~35,000 genes annotated in other sequenced diploid Solanaceae genomes.

Eggplant belongs to the genus *Solanum* and the family Solanaceae, which comprises over 3000 species adapted to a wide range of environments, including nine with sequenced genomes, i.e., potato^9^, tomato^10^, pepper^11,12^, tobacco^37^, petunia^38^, and four eggplants^13,16,17^ (*S. melongena*-HQ, *S. melongena*-NS, *S. melongena*-67/3, and *S. aethiopicum*). The Old World subgenus *Leptostemonum* comprises ~500 species and 30 sections, including half of the economically important crops^1^. The brinjal eggplant *S. melongena* belongs to section Melongena, whereas the closely related species, the scarlet eggplant *S. aethiopicum*, belongs to section Oliganthes. We found 6,087 gene families in common in the nine genomes, among which we identified 463 single-copy gene families (Fig. 3). *S. melongena* and *S. aethiopicum* diverged from each other ~2.4 Mya (Fig. S2). In addition, comparative genomics were performed among three sequenced eggplant genomes, *S. melongena*-HQ, *S. melongena*-67/3 and *S. aethiopicum*, and three types of variations (SNPs, indels and SVs) were characterized. As expected, *S. melongena*-HQ has significantly higher numbers of SNPs (22,092,994), indels (1,988,560) and SVs (7362) when compared with *S. aethiopicum* than compared with *S. melongena*-67/3 (Fig. 5). SVs consist of deletions and insertions that may result in divergent gene expression and phenotypes^39–42^. Interestingly, asymmetric SV accumulation was found in potential regulatory regions of protein-coding genes among the different eggplants, with more deletions than insertions between *S. melongena*-HQ and *S. aethiopicum*. In contrast similar insertion and deletion levels were observed between the two *S. melongena* genomes. This phenomenon has also been detected between two subgenomes of the allotetraploid peanut^42^. Overall the genome sequence of the linear eggplant HQ-1315 and comparative genomic information of *S. melongena* with that of related species allowed for the identification of genomic divergence at the whole-genome level, and the findings provide genomic tools for the improvement of agronomic traits in eggplant.

Stress resistance and fruit morphology (i.e., shape and color) are important traits during eggplant domestication that are vastly different among cultivated *S. melongena* varieties and closely related species. *S. aethiopicum* is mostly grown in tropical Africa, with outstanding disease resistance to various pathogens, such as *Fusarium* and *Verticillium* and is cross-compatible with *S. melongena*^43,44^. We identified 301 NBS-LRR genes in *S. melongena*-HQ and 250 NBS-LRR genes in *S. melongena*-67/3. As expected, *S. aethiopicum* has a higher number of disease resistance genes, with 436 genes involved in ten types. *S. melongena*-NS (Japanese eggplant) and *S. melongena*-67/3 (European eggplant) both have dark-purple fruits, with elliptical, oval or round shapes, whereas *S. melongena*-HQ has unusually linear-shaped fruits with a bright-purple color (Fig. 1). We constructed an F_2_ segregating population and performed QTL mapping analysis on eggplant fruit length using the *S. melongena*-HQ genome (Fig. 7). A QTL interval for fruit length was identified within a 71.29–78.26-Mb region on chromosome E03, with a 99% confidence interval. Gene prediction was conducted by homology comparison based on the syntenic relationship between eggplant and tomato, which yielded 11 homologous genes for fruit size on eggplant chromosome E03. Combining these results with the identification of the QTL region *FS3.1* in our previous study^30^, we propose that *Smechr0301963* (the ortholog from *S. melongena*-67/3 is *SMEL_003g182360*), a gene potentially orthologous to *SUN* gene family members, is a key candidate gene for regulating eggplant fruit length.

Eggplant research is far behind that of other Solanaceae crops (i.e., tomatoes, peppers, and potatoes) and important crops such as cucumber. For QTL mapping research, previous studies have often used tomato genomes for collinear comparisons because of the lack of high-quality eggplant reference genomes^25–27,45,46^. Our study provides a high-quality eggplant genome that has wide applications in eggplant genetics and genomics studies, such as marker development, gene detection and chromosome evolution. In the present study, we detected QTL hotspots based on published QTL mapping results and marker information^23–30^, with 210 markers associated with 71 traits anchored to the *S. melongena*-HQ reference genome (Fig. 8; Supplementary Table S10). We identified and summarized 26 QTL hotspots, providing a valuable reference and basis for further exploration of regulatory genes controlling important traits in eggplant.

## Materials and methods

### Plant materials, DNA extraction, and genome sequencing

The eggplant cultivar HQ-1315 was selected for whole-genome sequencing; it is a high-generation self-crossbred inbred line with elongated purple fruits. HQ-1315 is an important parental material derived from the Vegetable Institute of Zhejiang Academy of Agricultural Sciences. The HQ-1315 plants were grown in a greenhouse at Qiaosi of Zhejiang Academy of Agricultural Sciences (Hangzhou, China) under standard conditions. DNA was extracted from the young leaves of HQ-1315 for genome sequencing using DNA Secure Plant Kit (TIANGEN, China) and broken into random fragments. Four kinds of DNA sequencing libraries were constructed, including a 350-bp insert size library, Nanopore library, 10× Genomics library, and Hi-C library, according to the manufacturers’ instructions. The genome was sequenced using Illumina NovaSeq PE150 and Nanopore PromethION according to standard Illumina (Illumina, CA, USA) and Nanopore (Oxford Nanopore Technologies) protocols at Novogene.

To estimate the eggplant genome size, k-mer distribution analysis was used, and 17-nt k-mers were employed to determine abundance with 93.33 Gb of paired-end reads. SOAPdenovo software was used to splice and assemble the reads into scaffolds with 41-nt k-mers.

### Genome assembly and evaluation

We used wtdbg2 software^47^ to assemble the noncleaned raw reads from Nanopore sequencing according to the Fuzzy Bruijn Graph (FBG) algorithm. To derive each point, a 1024-bp sequence was selected from the reads, and the points were connected to construct the FBG figure using gapped sequence alignments. Finally, a consensus sequence was obtained. We polished the consensus sequence three times with Nanopore reads using Racon software^48^. The split size was 50, and the other parameters were set to defaults. Paired-end clean reads obtained from the Illumina platform were aligned to the eggplant assembly using BWA software^49^ (v0.7.17). Postprocessing error correction and conflict resolution of the assembly were performed using the Pilon tool with default parameters. The fragScaff software^50^ was applied for 10X Genomics scaffold extension. Linked reads generated from the 10X Genomics library were aligned to the consensus sequence of the Nanopore assembly to obtain long scaffolds. The consensus sequences were filtered, and only those with linked-read support were used for subsequent assembly. Then, clean Hi-C data were aligned to the primary draft assembly using BWA software v0.7.17^49^. SAMtools^51^ was utilized to remove duplicates and nonaligned reads, and only read pairs with both reads in the pair aligned to contigs were considered for scaffolding. Ultimately, 12 superscaffolds (pseudochromosomes) were assembled from corrected contigs using LACHESIS software^52^.

To evaluate the accuracy of the assembly, short reads were blast searched against the genome using BWA software^49^. CEGMA (http://korflab.ucdavis.edu/datasets/cegma/) was used to assess the completeness of the eggplant genome assembly, and BUSCO v4^53^ analysis was performed to further evaluate the assembled genome.

### Transcriptome sequencing and gene annotation

HQ-1315 plants were grown in a greenhouse at Qiaosi of Zhejiang Academy of Agricultural Sciences (Hangzhou, China) under standard conditions. RNA from five different tissues (root, stem, leaf, flower, and fruit) was extracted for next-generation transcriptome sequencing and full-length transcriptome sequencing using Illumina NovaSeq PE150 as an auxiliary annotation. Transcriptome read assemblies were generated with Trinity^54^ (v2.1.1) for gene annotation.

To optimize the gene annotation, RNA-seq reads from different tissues were aligned to genome fasta sequences using TopHat^55^ (v2.0.11) with the default parameters to identify exon regions and splice positions. The alignment results were then applied as input for Cufflinks^56^ (v2.2.1) with default parameters for genome-based transcript assembly. A nonredundant reference gene set was generated by merging genes predicted by three methods with EvidenceModeler^57^ (EVM, v1.1.1) using PASA^58^ (Program to Assemble Spliced Alignment) terminal exon support and including masked transposable elements as gene prediction input.

For ab initio gene annotation, Augustus^59^ (v3.2.3), GeneID^60^ (v1.4), GeneScan^61^ (v1.0), GlimmerHMM^62^ (v3.04), and SNAP^63^ were used in the automated gene prediction pipeline. Individual families of interest were selected for further manual curation by relevant experts. For structural annotation, ab initio prediction, homology-based prediction, and RNA-seq assisted prediction were used to annotate gene models.

### Repeat annotation

A combined strategy based on homology alignment and a de novo search was used in the repeat annotation pipeline to identify repetitive elements in the eggplant genome. Tandem repeats were extracted using TRF (http://tandem.bu.edu/trf/trf.html) by ab initio prediction. For homolog-based prediction, the Repbase TE library and TE protein database were employed to search against the eggplant genome using RepeatMasker^64^ (version 4.0) and RepeatProteinMask, respectively, with the default parameters. For de novo-based approach prediction, a de novo repetitive element database was built with LTR_FINDER^65^, RepeatScout^66^, and RepeatModeler^67^, also with default parameters.

### Homolog prediction

A total of five species were included in homolog prediction: *S. tuberosum*, *S. melongena*, *S. lycopersicum*, *C. annuum*, and *N. tabacum*. Sequences of homologous proteins were downloaded from NCBI and aligned to the genome using tBlastn^68^ (v2.2.26; *E*-value ≤ 1e − 5). The matching proteins were then aligned to the homologous genome sequences using GeneWise^69^ (v2.4.1) software to produce accurate spliced alignments, which were applied to predict the gene structure contained in each protein region.

### Functional annotation

The functions of protein-coding genes were assigned according to the best match by aligning the protein sequences against the Swiss-Prot database using Blastp^70^, with a threshold of *E*-value ≤ 1e^−5^. Protein motifs and domains were annotated by searching against the ProDom^71^, Pfam^72^ (V27.0), SMRT^73^, PANTHER^74^, and PROSITE^75^ databases using InterProScan^76^ (v5.31). GO IDs^77^ for each gene were assigned according to the corresponding InterPro entry. Protein functions were predicted by transferring annotations from the closest BLAST hit (*E*-value < 10^−5^) in the Swiss-Prot and NR databases. We also assigned a gene set to the KEGG pathway database^78^ (release 53) and identified the best matched pathway for each gene.

### Noncoding RNA annotation

tRNAs were predicted using tRNAscan-SE software^79^ (http://lowelab.ucsc.edu/tRNAscan-SE/). rRNAs were identified by alignment to the rRNA sequences of related species using BLASTN. Other noncoding RNAs, including miRNAs and snRNAs, were identified by searching against the Rfam database^80^ (release 9.1) using INFERNAL software^81^ (http://infernal.janelia.org/).

### Gene family construction and expansion/contraction analysis

Protein sequences predicted from the *S. melongena*-HQ eggplant genome and eight other sequenced Solanaceae genomes, *S. tuberosum*, *S. lycopersicum*, *S. melongena*-NS, *S. melongena*-67/3, *S. aethiopicum*, *C. annuum*, *P. inflata,* and *N. tabacum*, were used for gene family clustering. The gene set from each species was filtered according to the three steps described by Sun et al.^13^, with slight changes. The genes encoding proteins of fewer than 50 amino acids were filtered out. The gene families of the four eggplant genomes (*S. melongena*-HQ *S. melongena*-NS, *S. melongena*-67/3, and *S. aethiopicum*) were extracted for Venn diagram analysis to identify species-specific gene families in *S. melongena*-HQ. GO and KEGG annotation was performed to investigate the functions of those species-specific genes.

The expansion and contraction of gene families were analyzed by comparing family sizes between the MRCA and each of the nine sequenced Solanaceae genomes using CAFE^82^. The corresponding *p*-value for each lineage was calculated using conditional likelihoods, and families with a *p*-value of 0.05 were considered significantly expanded or contracted. The expanded and contracted genes were also analysed by GO and KEGG annotation.

### Phylogenetic analysis

MUSCLE^83^ (http://www.drive5.com/muscle/) was used to align single-copy genes from representative Solanaceae genomes, and the results were combined to generate a superalignment matrix. Using RAxML^84^ (http://sco.h-its.org/exelixis/web/software/raxml/index.html), a phylogenetic tree of the nine sequenced Solanaceae genomes was constructed with the maximum likelihood (ML) algorithm and 1000 bootstrap replicates. *P. inflata* was designated as the outgroup. To determine divergence times based on the phylogenetic tree, the MCMCTree program implemented in PAML5 software^85^ was used. Divergence time calibration information was obtained from the TimeTree database (http://www.time.org/).

### Detection of WGD events

Protein sequences from *S. melongena-HQ*, *S. aethiopicum*, *S. lycopersicum*, *S. tuberosum*, *C. annuum*, and *A. thaliana* were used for BLASTP (*E*-value < 1e−05) searches within or between genomes to identify syntenic blocks, after which syntenic blocks were searched using MCScanX (http://chibba.pgml.uga.edu/mcscan2/) software according to the locations of the genes and the blast results. Muscle multiple sequence alignment was performed on the paralogous genes in the syntenic blocks, and the results of the protein alignment were used as templates to generate CDS alignment results. Finally, 4DTv values were calculated according to the comparison results, and a frequency distribution diagram of the 4DTv values and gene pairs was drawn.

### Chromosome collinearity analysis

The CDSs of two species in the comparison group were compared with BLAST software (http://last.cbrc.jp/), and JCVI was employed to locate syntenic blocks and map them with the following parameters: —cScore=0.9, —minspan=30, (https://github.com/ tanghaibao/jcvi/wiki/MCscan- Python - version).

### Identification of SNPs, indels, and SVs

The genome sequence of *S. melongena*-HQ was aligned to that of *S. melongena*-67/3 and *S. aethiopicum* using BWA v0.7.17^49^ using default parameters. Picard tools v1.9.4 (https://broadinstitute.github.io/picard/) was applied to sort the alignment result sequence alignment map (SAM) files. SNPs and indels were called using Genome Analysis Toolkit^86^, and related genes were called according to genome position using an in-house Perl script.

Clean reads of *S. melongena*-HQ were aligned to those of *S. melongena*-67/3 and *S. aethiopicum* using BWA v0.7.17^49^ with default parameters. BreakDancerMax-0.0.1r61 was used for genome-wide detection of SVs with default parameters^87^. Deletion and insertion structure variations <10 bp or >10 kb in length were discarded. For the identification of SV genes, any gene with SVs in the main body or upstream/downstream regions was defined as an SV gene; otherwise, it was defined as a non-SV gene.

### Identification of the NBS gene family and transcription factors

Most NBS-encoding genes in eggplant were identified based on NB-ARC (NBS) conserved domains that are shared within the gene family and have relatively conserved NBS domains. The latest Markov model for the NBS transcription factor PF00931 was downloaded from the Pfam database (http://pfam.xfam.org/). The HMMER program was used to search for proteins containing this domain against the annotated protein database using the PF00931 domain as a query, with a cutoff *E*-value of 1e−4. To annotate the maximum number of NBS genes in the genomes, we also used the obtained NBS protein sequences for homologous annotation of genome sequences. tBlastn was applied for homology comparison, and the upper and lower segments of the comparison region were expanded by 5 kb each. Genewise software was then used for gene structure prediction, and homologous protein sequences were screened with PF00931. For the identification of transcription factors, iTAK-1.5-alpha software was utilized to predict transcription factors among the longest transcribed translated protein sequences of each species.

### QTL-seq

An F_2_ population with 129 individuals was generated from a cross between HQ-1315 (linear-long fruits) and 1815 (round fruits), and phenotypic data on eggplant fruit length (L), diameter (D) and fruit shape index (L/D) were collected. Three mature fruits of each individual plant were selected for measurement; plants with extremely long/short fruits were selected and pooled according to the fruit length statistics. Equal amounts of DNA from the young leaves of 20 extreme individuals in each pool were mixed and sequenced. GATK 3.8 software was used to improve multiple-sample SNP and indel detection, and VariantFiltration was applied for filtering^86^. The SNP index was calculated with QTL-seq^88^ methods. Indel markers that were exactly the same as those of the parent were assigned an indel-index of 0, with those completely different from the parent assigned an indel-index of 1. To intuitively reflect the distribution of all indices on the chromosome, the SNP index and indel index were combined to obtain Δ(all-index). Any interval with an aΔ(all-index) value higher than the threshold at the 95% confidence level was selected as a candidate interval. SNPs and indels were annotated using ANNOVAR^89^.

## Supplementary information

Fig. S1

Fig. S2

Fig. S3

Fig. S4

Fig. S5

Table S1

Table S2

Table S3

Table S4

Table S5

Table S6

Table S7

Table S8

Table S9

Table S10
