# Supplementary figures and images for: A high-quality chromosome-level genome assembly reveals genetics for important traits in eggplant
Source: Hortic Res. 2020 Sep 21;7:153. doi: 10.1038/s41438-020-00391-0 (PMC7506008; doi:10.1038/s41438-020-00391-0)

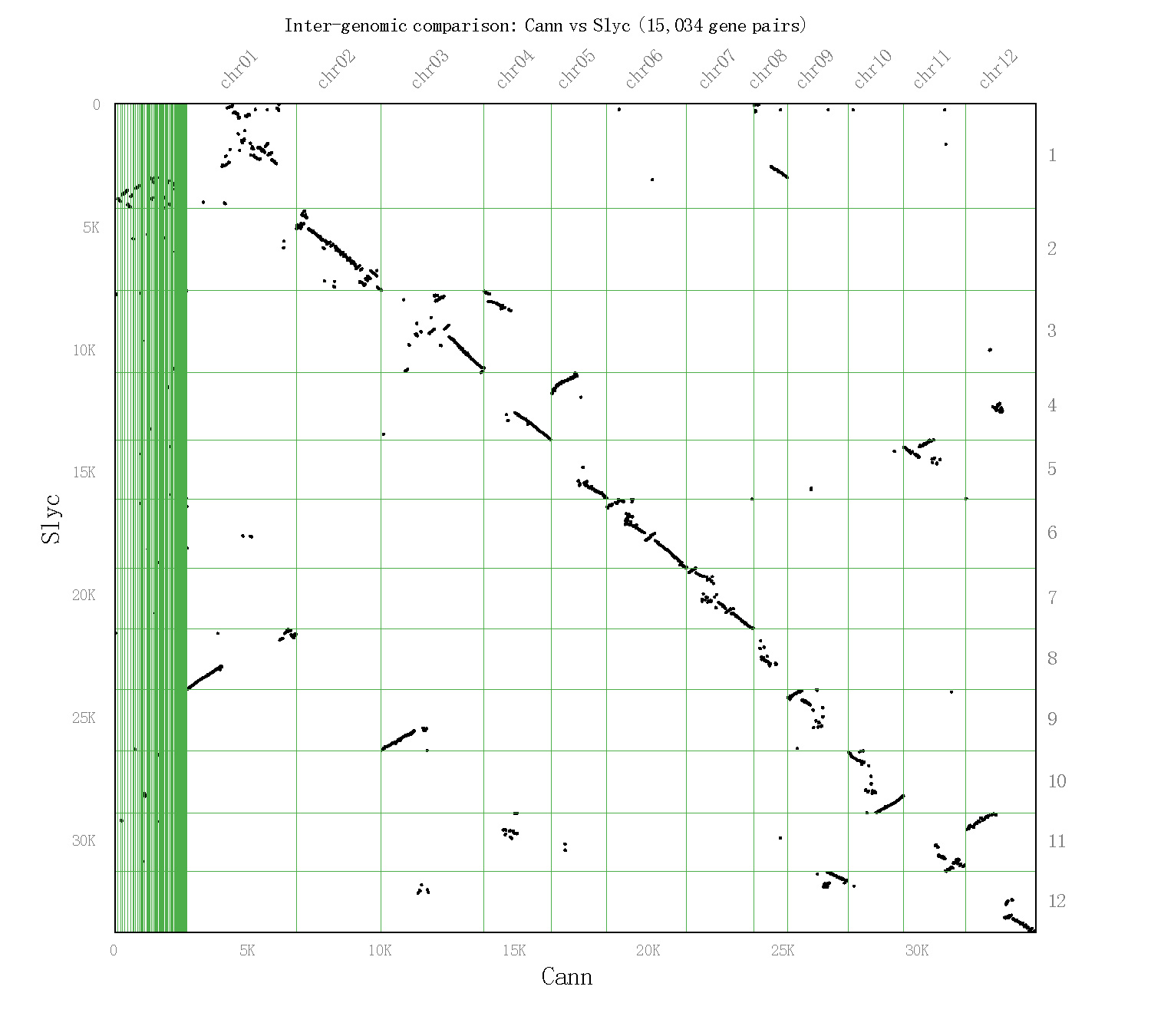

Supplement: Supplementary file 1 — Fig. S1 [file 41438_2020_391_MOESM1_ESM.jpg]

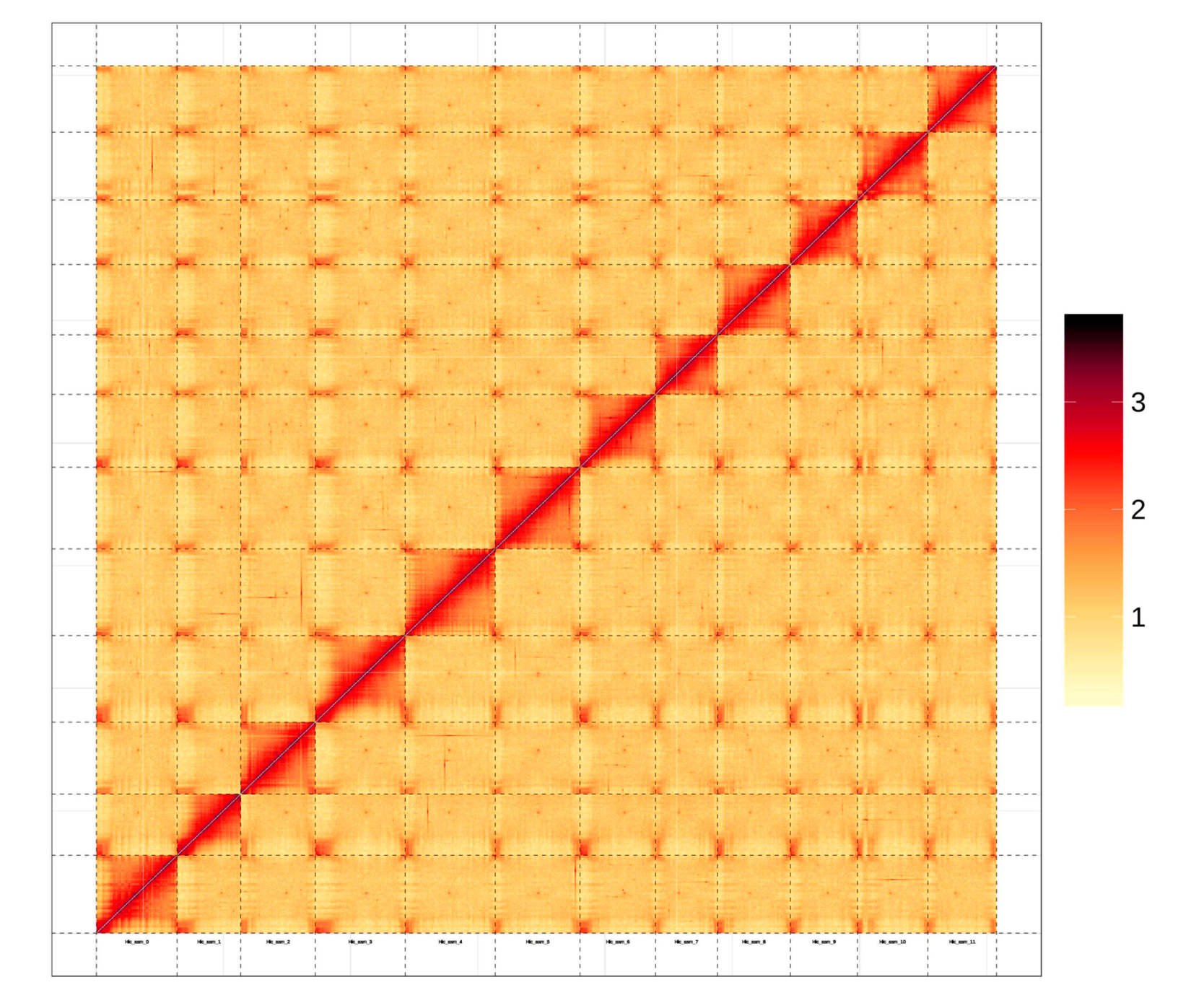

Supplement: Supplementary file 2 — Fig. S2 [file 41438_2020_391_MOESM2_ESM.png]

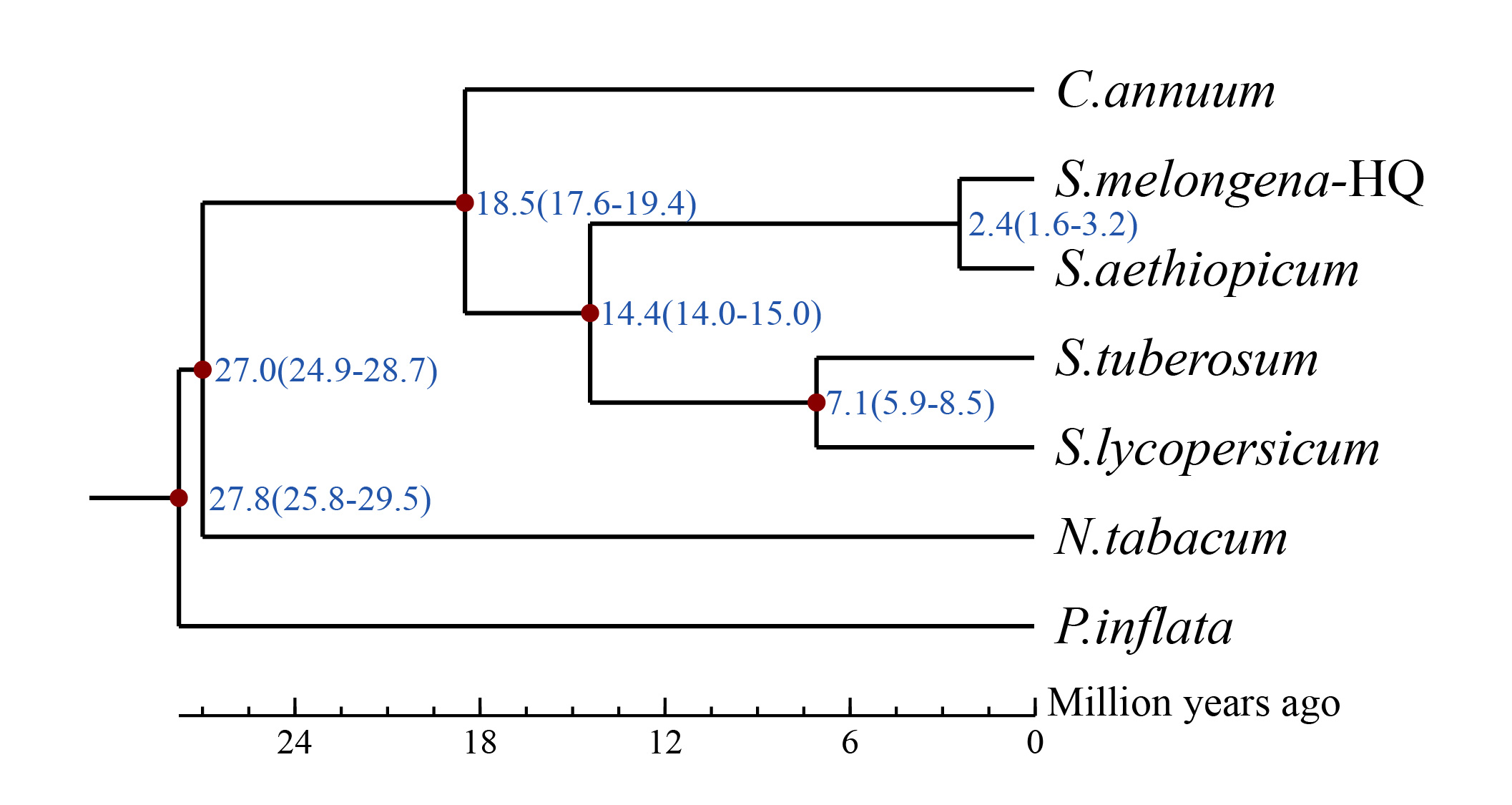

Supplement: Supplementary file 3 — Fig. S3 [file 41438_2020_391_MOESM3_ESM.jpg]

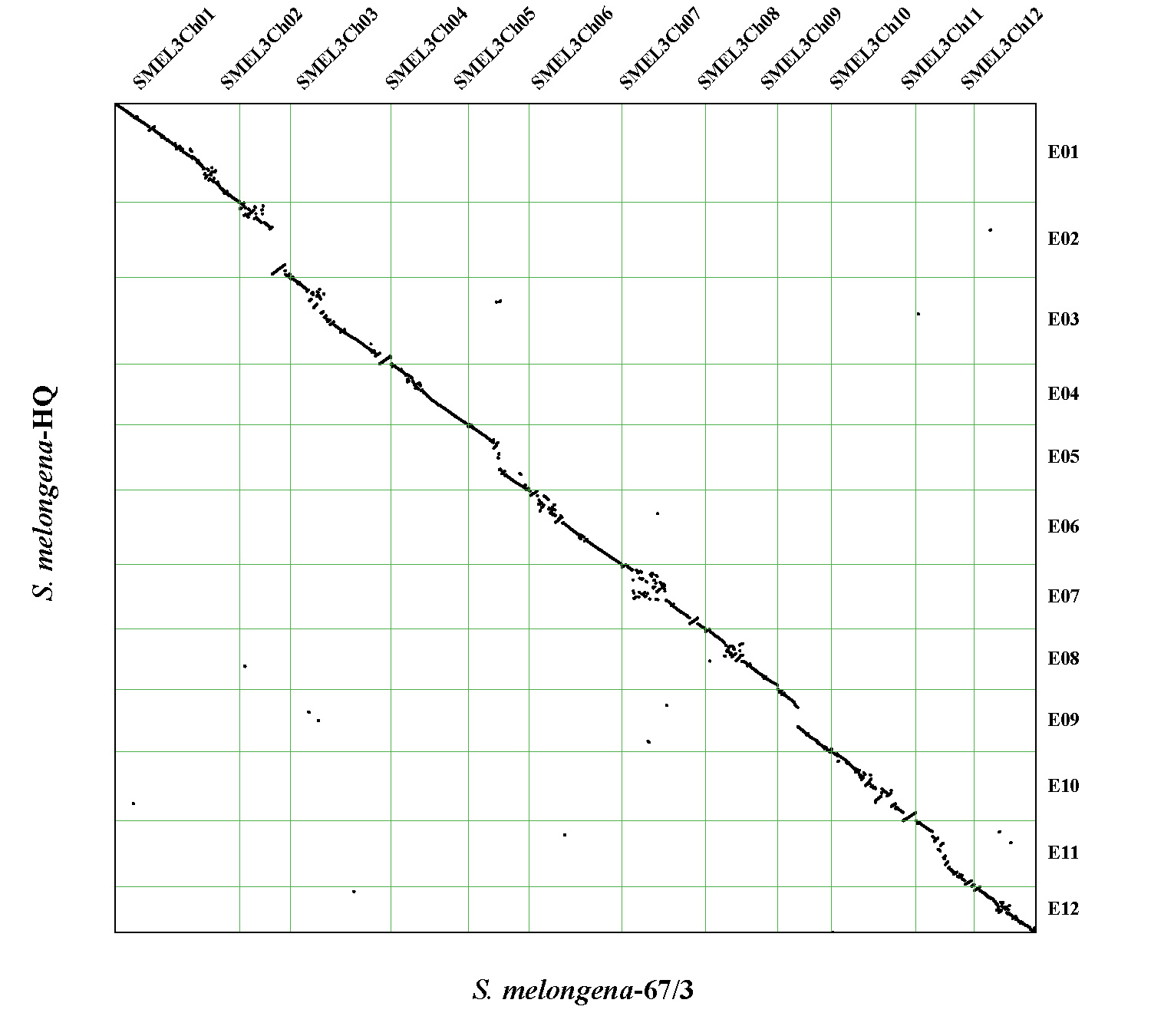

Supplement: Supplementary file 4 — Fig. S4 [file 41438_2020_391_MOESM4_ESM.jpg]

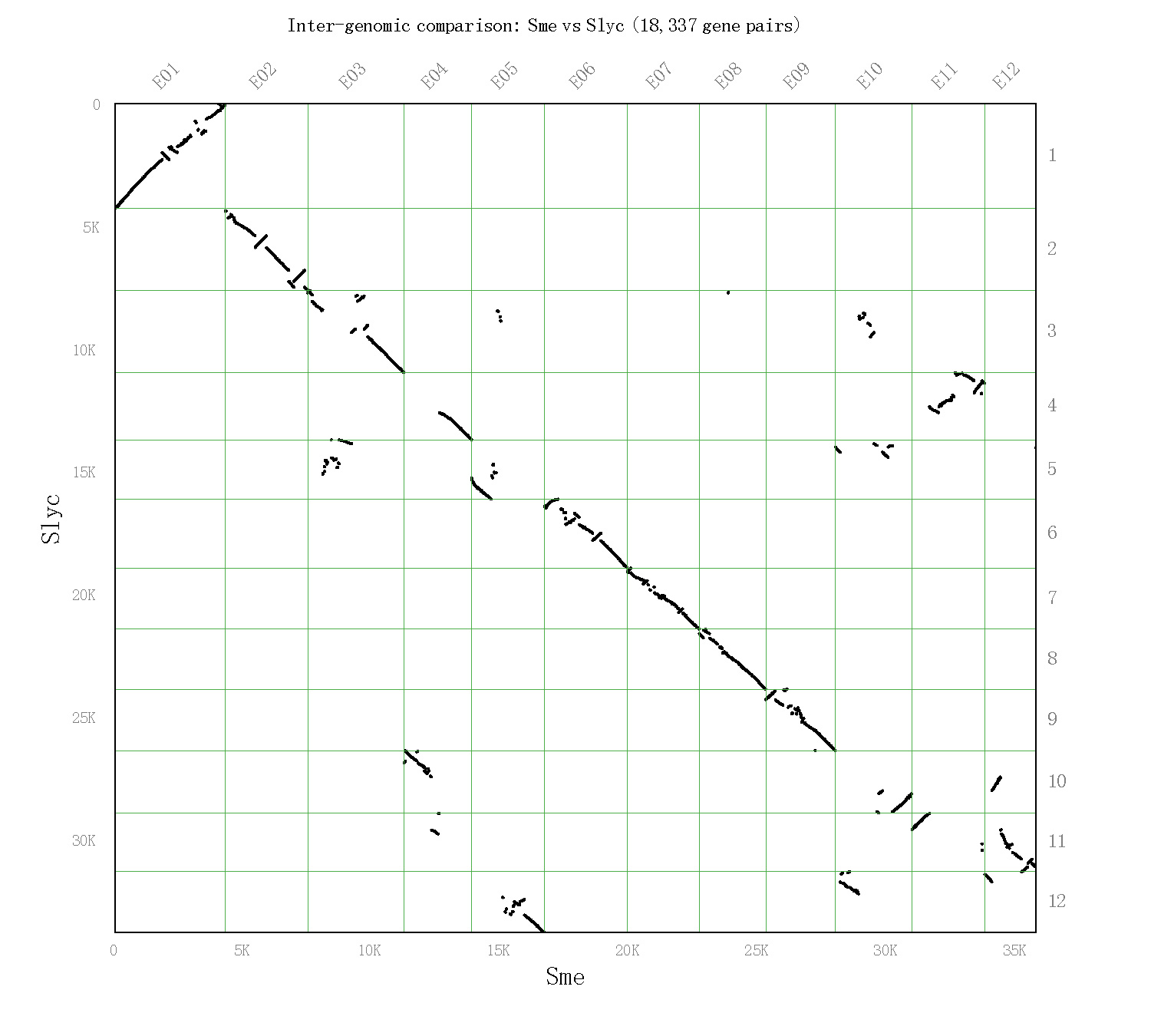

Supplement: Supplementary file 5 — Fig. S5 [file 41438_2020_391_MOESM5_ESM.jpg]
